# A user-generated data based approach to enhancing location prediction of financial services in sub-Saharan Africa

**DOI:** 10.1016/j.apgeog.2019.02.005

**Published:** 2019-04

**Authors:** Grant McKenzie, R. Todd Slind

**Affiliations:** a Department of Geography, McGill University, Canada; b Spatial Development International, Seattle, USA

**Keywords:** Financial access, User-generated content, Social media, Kenya, Random forest

## Abstract

The recent increase in user-generated content and social media adoption in developing countries offers an unprecedented opportunity to better understand the accessibility and spatial distribution of financial services in sub-Saharan Africa. Financial inclusion has been identified as a priority by multiple agencies in the region and on-the-ground efforts are currently underway to identify previously unknown financial access points in numerous developing African countries. Existing techniques for estimating the location of these access points rely on spatial analysis of often outdated or unsuitable publicly available datasets such as population density, road networks, etc., as well as expensive and time consuming surveys of locals in the region. In this work we propose an approach to augment existing spatial data analysis techniques through the inclusion of user-generated geo-content and geo-social media data. Through a comparison of standard regression models and machine learning techniques, this work proposes the use of alternative data sources to build prediction models for identifying financial access locations in countries where current estimation models are insufficient. With a better understanding of geospatial distribution patterns this work aims at reducing data acquisition costs and providing decision makers with critical data more quickly and efficiently. Finally, we present a mobile application built on the outcomes of this analysis that is currently being used to better inform on-the-ground data collection efforts.

## Highlights

• Addressing concerns over financial inclusion in sub-Saharan Africa is important.
• Information on accessibility and spatial distribution of mobile money agents is limited.
• User-generated content & geo-social media usage is growing in sub-Saharan Africa.
• We augment existing spatial analysis techniques through inclusion of these data.
• A prediction layer was developed for inclusion in our mobile data collection application.

## 1 Introduction

**Fig. 1:**
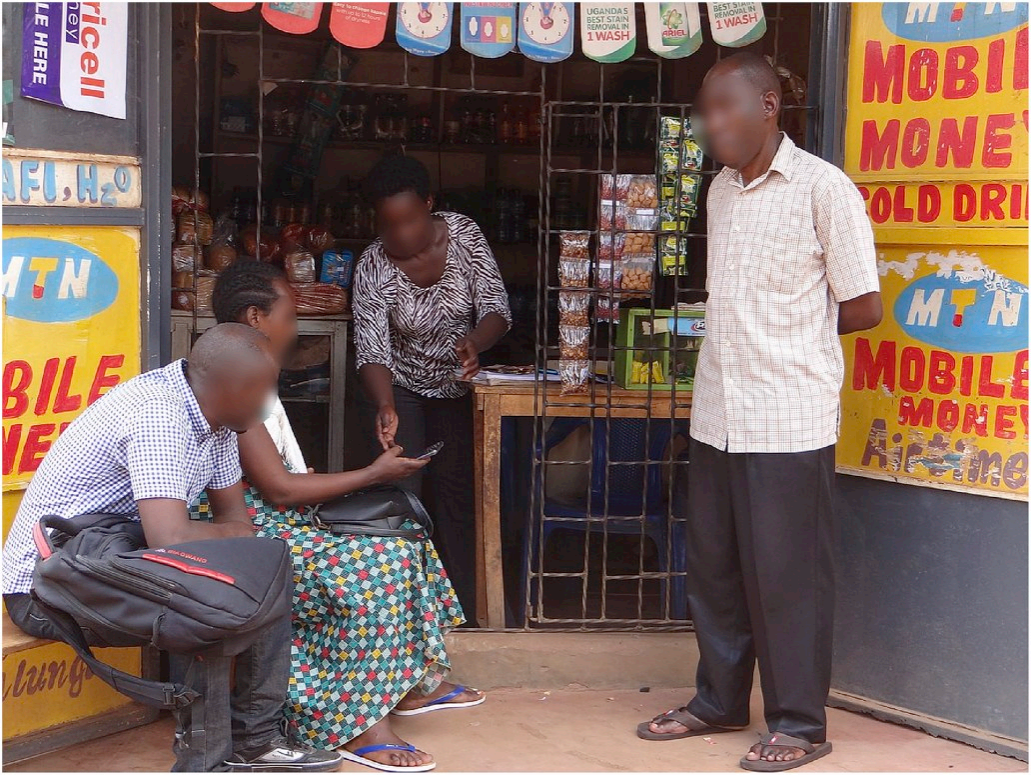
An example of a mobile money provider in Uganda. Source: Wikimedia Commons. License: CC 4.0.

By current estimates, the number of individuals in sub-Saharan Africa (SSA) with bank accounts at formal financial institutions is 25% (European Investment Bank, 2016), a number that has remained relatively stagnant, growing by only a couple of percentage points over the past four years (Triki & Faye, 2013). By comparison, mobile money accounts in East African countries, especially Kenya and Tanzania, have increased dramatically. The term mobile money here represents the use of mobile devices to transfer money between users, pay bills, or purchase items. Mobile money providers are those companies through which an individual deposits or withdraws local currency to or from their mobile money account. Mobile money providers are typically fixed-location, corner stores to which a customer can go to exchange currency for mobile money (see Fig. 1 for an example). Safaricom, a leading Kenyan mobile network operator, launched a mobile device-based payment system called M-Pesa in 2007 that revolutionized financial transactions across much of East Africa. In 2016, it was estimated that mobile device penetration in Kenya surpassed 90%, an increase of over 6% in one year (C. A. of Kenya, 2016). And while only a small portion of the Kenyan population have traditional bank accounts, over 58% percent of individuals in Kenya use mobile money (World Bank, 2015) to transfer funds between people and/or businesses or borrow money by way of a loan (Ochieng, 2016). Mobile money has such a dominant role in the Kenyan economy that in 2014 M-Pesa, by far the leading mobile payment system, accounted for over 60% of the country's gross domestic product (Economist Intelligence Unit, 2014).

While the rise of mobile money has shown to reduce poverty rates (Suri & Jack, 2016) and increased gender equality in many developing nations (Sekabira & Qaim, 2017), there are concerns over economic impact (Suri, 2017), taxation (Olingo, 2016), and the influence of a single mobile network operator. The external focus on the striking growth in usage of mobile money has also served to magnify the financial divide within the country. During the FinAccess 2014 conference Njuguna Ndung'u, Governor of the Central Bank of Kenya, gave a keynote address in which he encouraged the expansion of financial inclusion in Kenya (Ndung'u, 2014). In this keynote, Professor Ndung'u reiterated that while a considerable portion of the Kenyan population has access to mobile money infrastructure, a quarter of the population remains entirely excluded. With the goal of increasing financial inclusion, the Central Bank of Kenya, specified that a first step should include the identification of all Financial Touch Points (FTP)^1^ within the country. While there are on going efforts to collect location information on FTP providers in Kenya (Brand Fusion, 2015a; FSD Kenya, 2015), the turn-over rate and movement of providers within the country are high. In actuality, the locations of many FTP are still not known.

Efforts to better understand the distribution of financial services in Kenya are on-going. These are focused on the spatial distribution of mobile money infrastructure to identify opportunities for business expansion, agricultural services, etc. (Hughes & Lonie, 2007; Kim, 2016; Kirui, Okello, Nyikal, & Njiraini, 2013). On-the-ground data collection efforts continue in SSA regions with the Humanitarian OpenStreetMap Team (Uithol, 2015) following other teams such as Brand Fusion (Brand Fusion, 2015a) in their data collection efforts. Most of these on-the-ground efforts involve canvassing entire countries on motorcycles with GPS units in an attempt to identify new FTP locations or view the identification of FTP as a secondary goal to mapping a country. Collectors focus their efforts on highly populated regions, surveying locals and known FTP providers (Brand Fusion, 2015b). In general though, there is a lack of informed strategy on where to look for these financial touch points in the most efficient manner. Population density maps and local knowledge are an important step and our goal is that the methods proposed in this work can be used to augment existing ones. To this end, this work aims to build a model for predicting the location of financial touch points based not only on population densities, but other publicly available datasets, both traditional authoritative (e.g., land use, school locations) and user-contributed (e.g., volunteered information and social media).

In the last year, the number of smartphone users in SSA has grown substantially. The percentage of users in Kenya with smartphones was roughly 44% in 2016, a substantial shift from the previous year of 27% (Xylouris, 2016). This growth in smartphone access has also given rise to a substantial increase in social media usage. Recent reports show social media usage at 58% of the most popular activities conducted with a mobile device followed by search engines at 39% and email at 30% (Xylouris, 2016). Facebook, one of the most popular social media platforms in the world has recently focused their attention on SSA as a region for expansion (Facebook People Insights, 2017). These efforts are paying off with recent statistics showing that 170 million Africans have joined Facebook, most of which connect through their mobile device (Shapshak, 2017). Of these, 6.1 million are from Kenya. (B. A. of Kenya, 2016). Twitter, has also seen an increase in adoption with monthly active users counted at roughly 2.2 million (Kaigwa, Madung, & Costello, 2015). As users interact with these platforms, they contribute significant amounts of digital content. This content ranges from photographs and opinions to restaurant reviews and group chats. The fact that much of this interaction happens via mobile device is of importance as well. Many smart devices contain high resolution location sensors such as GPS or Wi-Fi and social media applications make use of this information which lead to social contributions that contain geographic data such as places, local businesses and geotagged social posts. Through the various application programming interfaces (APIs) offered by these platforms, researchers now have access to much of this published content. The resolution of these data both spatially and temporally offer unique insight into the behavior of individuals within the region. Not only can these data be used to enhance low resolution (and often outdated) population density maps but contributions such as those that mention local businesses can be used to better predict the location of previously unmapped entities, such as mobile money providers and other FTP.

Social media data are often defined as a subcategory of user-generated content (UGC), one that may contains geographic information, but is often not contributed explicitly with the geographic content in mind (McKenzie & Janowicz, 2014). Another source of UGC common to the geography domain is volunteered geographic information (VGI) (Goodchild, 2007). One of the popular platforms for this type of information is OpenStreetMap,^2^ a rich set of geospatial data contributed to, and curated by, thousands of citizens worldwide. In recent years there have been substantial efforts to increase coverage and quality of geographic data and maps in SSA.^3^ These data in many cases are more up-to-date and have greater coverage than many government or commercial geographic datasets and knowing this, we propose their inclusion in our approach to predicting financial access location in Kenya.

### 1.1 Research contribution

The purpose of this work is to develop a method for predicting financial touch points in Kenya. Specifically, we are interested in determining if at least one FTP can be identified within a specific set of grid cells. Building on traditional authoritative datasets, we examine the fitness of emerging data sources for inclusion in an FTP prediction model and ultimately as a layer in a mobile application for data collection. To this end we address the following four research questions (RQ).

RQ1 With the goal of identifying financial touch points in Kenya, how do geo-tagged social media and volunteered geographic information fare in comparison to authoritative datasets? To address this question, we explore the distribution and correlation of various datasets with known FTP in Kenya. We report on the accuracy of using these data independently for estimating FTP counts and locations.

RQ2 Can social media data and volunteered geographic information be used in combination with existing authoritative datasets to produce better FTP prediction models than those generated from the datasets independently? Here we examine two traditional regression methods, namely ordinary least squares and spatial lag as well as two machine learning regression approaches, namely support vector regression and random decision forest (RDF). The accuracy of these models are reported via three measures.

RQ3 Provided a best fit model, can we validate this approach through on-the-ground identification of previously unknown FTP? Secondly, how accurate is the best fit model in identifying FTP in Kenya's neighboring country of Uganda? We assess and report on the accuracy of the model and identify important differences between the two countries that likely impact the accuracy of the model.

RQ4 Can the FTP prediction model provide the foundation of a mobile application for FTP data capture and validation? We present a prototype mobile application currently employed by users on-the-ground to add, edit and delete FTP locations, driven by an FTP prediction layer generated from our best fit model.

The remainder of this article is organized as follows. In Section 2 we discuss existing research related to the topic and methods, and in Section 3 we present the various datasets used in this work. The methods used in predicting financial touch points are given in Section 4, with the results of the analysis shown in Section 5. Two different approaches for validating the data set are presented in Section 6 with an overview of the mobile application in Section 7. Finally, conclusions and next steps are stated in Section 8.

## 2 Related work

Existing work in this area has highlighted the importance of understanding mobile financial services in sub-Saharan Africa specifically as it relates to poor populations (Porter, 2012; Tanle & Abane, 2017). Some of this research has used data collected directly from mobile devices (Dillon, 2012) while others have focused on the broader impact of the technology (Asongu & Nwachukwu, 2016). Mobile money usage is not unique to sub-Saharan Africa. Many other countries have adopted mobile money systems, China being one of the leading proponents of the technology (Guo & Bouwman, 2016). Recent reports have shown that payment systems suck as Alipay and WeChat pay are having significant impacts in shaping the country's economy (Armstrong & Wang, 2018). In recent years, the focus has shifted from the availability of mobile devices to the actual usage patterns and applications. Short messaging service (SMS) and social media usage have grown substantially and are having a sizable impact on the developing world for everything from political movements (Howard & Parks, 2012) to monitoring and tracking health epidemics (e.g., Ebola) (Wesolowski et al., 2014).

As social media usage and user-generated content grows in developing countries, so does that availability of geotagged content (Stefanidis, Crooks, & Radzikowski, 2013). The development of crowd-sourcing crisis tools such as *Ushahidi* (Okolloh, 2009) and *Missing Maps* (Palen, Soden, Anderson, & Barrenechea, 2015) have successfully demonstrated that geotagged social content can have a substantial impact during crisis relief efforts. Recent work by Adams, McKenzie, & Gahegan (2015) has also shown that user-generated geo-tagged content from travel blogs and Wikipedia articles can be used to identify thematic regions around the world further emphasizing the power of crowd contributions. Existing work by Linard et al. (2014, pp. 1–16) has examined the inclusion of volunteered geographic information in enhancing the WorldPop dataset. Their efforts demonstrated that OpenStreetMap vector data can be used to combination with satellite imagery to further refine global population estimates. Further work has used a combination of VGI-based gazetteer data and social media ‘check-ins’ to determine citizen locations (McKenzie & Janowicz, 2015) and prioritize evacuation zones (Hu, Janowicz, & Couclelis, 2017).

From a methodological perspective, machine learning regression models have been quite successful in a variety of scenarios. The range of literature in this area speaks to the complexity and variety of models. Previous work on the role of spatial autocorrelation in standard regression (Anselin, 2001) is making it's way into machine learning (e.g., SVM, RDF, etc.) discussions (Cracknell & Reading, 2014). Existing work from Song, Kwan, Song, & Zhu (2017) compared spatial econometric models to a random decision forest approach in modeling fire occurrence and demonstrated the benefits and disadvantages of the different approaches. Stevens, Gaughan, Linard, & Tatem (2015) employed a RDF model in disaggregating census data for population mapping with the goal of enhancing the WorldPop dataset and recent work on identifying landscape preferences determined that an RDF approach applied to Flickr photos produced the best results (Chesnokova, Nowak, & Purves, 2017).

## 3 Data

In this section, we provide an overview the datasets used in constructing the FTP identification models. The financial touch points are introduced as well as the predictors classified as VGI, Social Media, and Authoritative datasets.

### 3.1 Financial touch points

**Fig. 2:**
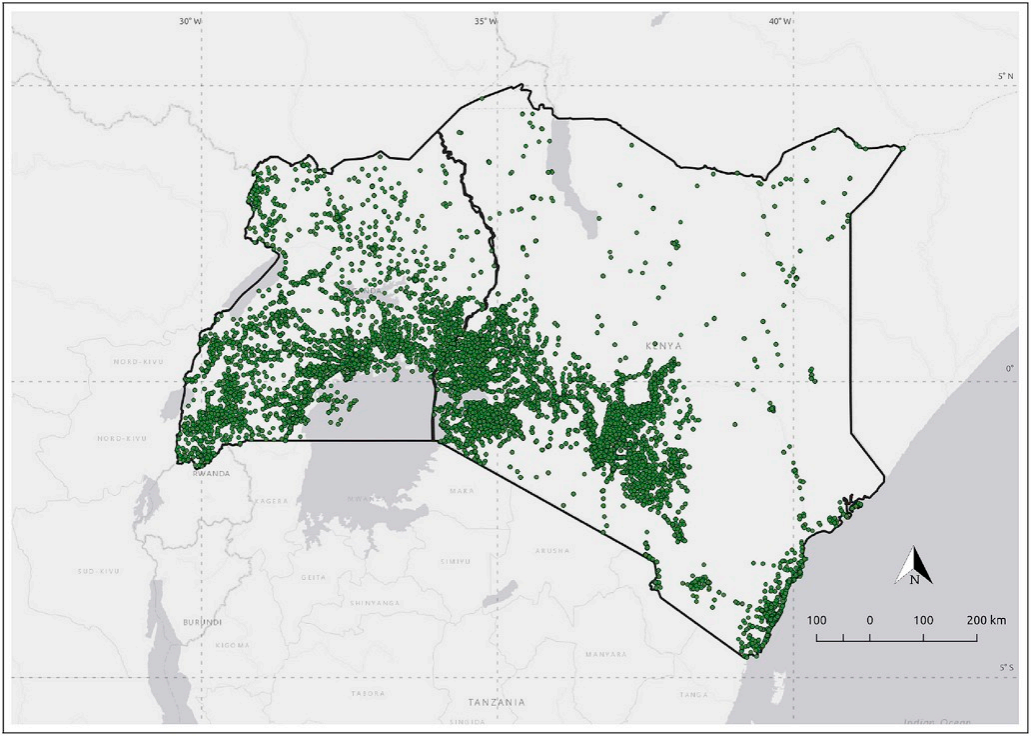
Financial Touch Points (FTP) in Kenya (83,273) and Uganda (45,417). Base map by ESRI.

On-the-ground data collection efforts by *Brand Fusion*^4^ resulted in a dataset of verified FTP in Kenya (Brand Fusion, 2015a). Brand Fusion estimates that these data, collected in 2015, represent a high portion of all FTP within Kenya but the data are non-exhaustive as FTP may have been missed by data collectors, locations may have been established since the last round of data collection, or FTP may have moved. The purpose of this paper in this case is to use geospatial indicators near to these known FTP to predict and identify previously unidentified FTP in Kenya. This 2015 Brand Fusion dataset identified 83,273 FTP in Kenya and these form the basis on which our prediction model is trained and tested. Fig. 2 shows the distribution of these FTP in Kenya as green markers. The Humanitarian OpenStreetMap Team (HOT) collected FTP for neighboring Uganda (Uithol, 2015). In total, 45,417 verified FTP were identified in Uganda and these points will form the basis of our follow-on analysis. Visually, the highest density of FTP appear to occur in densely populated regions around Nairobi, Nyanza (Kenya), Kampala and Mbarara (Uganda). Spatial analysis of these FTP locations through *Moran's I* (Moran, 1950) and *Ripley's K* (Ripley, 1976) functions confirm this, indicating clear spatial clustering within these datasets. While the high population areas show the highest numbers of FTP, it is the rural regions that are of particular interest to government and non-government agencies.

### 3.2 Predictors

We compare and contrast a number of different datasets from a wide variety of sources with the purpose of determining how the inclusion of these data aid in predicting FTP locations. Table 1 lists these datasets along with their sources and our assigned category tag. These categories consist of two types of user-generated content, namely *volunteered geographic information (VGI)* and *social media (SM)* as well as more traditional datasets which by comparison we label *authoritative (AUTH)*.

**Table 1:** Datasets used in identifying financial touch points.

Dataset Description	Source	Year	Category
Estimated persons per 3 arc-second (roughly 100 m) cell	Worldpop	2015	AUTH
Primary & Secondary School Locations	OpenAfrica	2015	AUTH
0.5 km MODIS-based Global Land Cover Climatology	USGS	2014	AUTH
Global Rural-Urban Mapping Project (GRUMPv1)	NASA	2011	AUTH
GeoNames Places	GeoNames	2016	AUTH
LandScan-based Populated Places	Natural Earth	2016	AUTH
OSM Roads	OpenStreetMap	2016	VGI
OSM POI	OpenStreetMap	2016	VGI
Facebook Places	Instagram API	2016	SM
Tweets	Twitter API	2016	SM
Foursquare Venues	Foursquare API	2016	SM

#### 3.2.1 Authoritative datasets

We define the *authoritative datasets* in this work as those not created through direct citizen contributions or social media data extraction. These datasets were generated using more authoritative and controlled mechanisms and are therefore, allegedly, less prone to user bias or classification error. These data have been used in numerous other studies in estimating everything from population density and land use to human mobility and predicting disease outbreak (Friedl et al., 2002; Linard, Gilbert, Snow, Noor, & Tatem, 2012; Ruktanonchai et al., 2016; Wesolowski et al., 2014).

The 2015 *WorldPop* data contains high resolution (∼100 m cell size) human population distribution estimates. The data was generated from a combination of remote sensed imagery, census and existing geospatial datasets (e.g., road networks) (Deville et al., 2014; Stevens et al., 2015). The Socioeconomic Data and Application Center in NASA's Earth Observation System Data and Information System group produces the *Global Rural-Urban Mapping Project (GRUMP)* data. Similar to the WorldPop dataset, these data are produced through a combination of census and satellite data (including night-time lights) at a resolution of roughly 1 km. Version 1 of this dataset was produced in 2011 and provides rural and urban population density estimates for the year 2015 (Balk et al., 2006; Freire, Kemper, Pesaresi, Florczyk, & Syrris, 2015). *Urban land cover type* regions were also extracted from the 0.5 km MODIS-based Global Land Cover Climatology dataset (Broxton, Zeng, Sulla-Menashe, & Troch, 2014) generated in 2014.

Primary and Secondary *school* locations were accessed from OpenAfrica, a web portal for open data in African countries. School locations for Kenya were most recently updated in 2015 and contributed by the Kenya Open Data Initiative  (Rahemtulla et al., 2012). Similarly, school locations for Uganda were collected by the Uganda Bureau of Statistics and the Ministry of Education and Sports from 2004 to 2010. Places were downloaded from the *GeoNames* placename gazetteer which is made up of a number of sources, most notably the National Geospatial-Intelligence Agency and the U.S. Board on Geographic Names for regions outside of the United States. This point data represents everything from mountain tops to water wells. Natural Earth *Populated Places* data were used in this research which is based on LandScan-derived population estimates  (Dobson, Bright, Coleman, Durfee, & Worley, 2000). Natural Earth devised the dataset based on regional significance of places over population census, differentiating it from the grid-based systems previously mentioned  (Natural Earth, 2014). Counts of these datasets are shown in Table 2.

**Table 2:** Counts for the predictor datasets in Kenya and Uganda. Note that both the WorldPop and GRUMPv1 data are not count based datasets and so are not reported here.

Dataset	Kenya	Uganda
Facebook Places	8107	4377
Twitter Tweets	204538	156426
Foursquare Venues	4016	2075
OpenStreetMap POI	16739	44203
OpenStreetMap Roads (km)	98381	48676
Schools (Primary & Secondary)	37317	29372
GeoNames Places	26038	25978
NE Populated Places	56	42

#### 3.2.2 User-contributed data

User-contributed data are those created either via volunteered geographic information (VGI) means or social media (SM) contribution. Typically contributions to these data are made from non-experts and do not rely on statistical models built from existing data sources. Anyone can add a place, venue, road, or post (tweet) to one of these datasets without requiring secondary approval.^5^

#### 3.2.3 Volunteered geographic information

*OpenStreetMap* Points of Interest were downloaded for Kenya using the OsmPoisPbf extraction tool.^6^ Table 2 lists the total number of POI with roughly 2% (339) of these being tagged as *MONEY BANK* or *MONEY EXCHANGE*. On examination of these tagged POI, the overwhelming majority of these were brick-and-mortar bank branches with few mobile money providers or lenders. These mobile money providers and lenders are either corner stores/grocers or dedicated shops (e.g., M-Pesa). The OpenStreetMap Road data was also extracted in January 2016 and consists of high resolution road network data contributed by volunteers. These data are notably of a higher resolution and wider spatial coverage than the road network datasets available from the Kenyan government GIS web portal.

#### 3.2.4 Social media data

Social media data for this research involved three sources of geotagged content. Instagram and Foursquare both have digital gazetteers of place locations contributed by individuals while twitter allows contributors to geotag their posts with geospatial coordinates.

The Instagram locations API^7^ was used to extract Points of Interest for Kenya. Instagram uses *Facebook Places* as its gazetteer, with the purpose of allowing individuals to tag their photographs with a place name. Their API offers limited access to this gazetteer. In total, 8107 places were accessed in Kenya. The *Twitter* Streaming API^8^ was used to access geotagged tweets within Kenya over a 5 month time span from January through May 2016. Only those tweets that included precise geographic coordinates and sourced from the *Android Twitter App* or *iPhone Twitter App* were employed here. In this work, only the geographic location of the tweets was relevant for this research though future work may explore the content and language variation within the text of the tweets. The *Foursquare Venues* Search API^9^ was employed to access Points of Interest in the Foursquare gazetteer. Foursquare began curating POI in March of 2009 and has been more transparent in how they collect places (Perez, 2013) than Facebook. Notably Facebook has a much larger user-base (2 billion vs. 45 million) however.

## 4 Methods

To start, a spatial grid was generated over the entire country of Kenya at a resolution of 0.02°, or approximately 2.2 km at the equator. Selection of this resolution was based on trade off between reasonable travel time within each grid (for on-the-ground collection efforts and actual FTP users) and reduced computational complexity. This resulted in 120,111 grid cells across Kenya. The grid was intersected with the FTP data producing an FTP grid layer with aggregated count cells ranging in value from 0 to 2402 (in Nairobi). Similar layers were constructed for each of the predictor variables using the same grid bounds and resolution. Finally, each gridded layer was normalized to between 0 and 1. This was to ensure that each variable could be compared to one another without one predictor overpowering the others. While not essential in a linear or spatial regression model, it is particularly important for a random decision forest approach (Gislason, Benediktsson, & Sveinsson, 2006).

### 4.1 Individual predictors

The goal in the initial analysis for RQ1 is to determine how accurate each individual dataset is in identifying FTP. We first examine the correlation between each gridded dataset and the gridded FTP layer. Table 3 shows the Spearman's correlation matrix of all predictors. Notably, all datasets show positive correlation with the number of FTP per cell. The Worldpop, Grump and School datasets show the highest correlation with Facebook Places also showing a reasonably high value. Interestingly tweets have a relatively low correlation with FTP (0.11) and an even lower correlation with the other social media/user-generated content datasets (e.g., 0.05, 0.02) indicating that there is little similarity between our social media places and the geotagged tweets. On the other hand, GRUMP data are highly correlated with the WorldPop dataset.

**Table 3:** Spearman's correlation between Kenya dataset cell counts. All p<0.01.

	FTP	Facebook	Foursquare	Twitter	OSM POI	OSM Roads	Schools	GRUMP	WorldPop	Land. Urban	GeoNames	NE
FTP	1.00	0.50	0.29	0.11	0.33	0.08	0.50	0.55	0.57	0.38	0.27	0.29
Facebook Places	0.50	1.00	0.38	0.05	0.13	0.14	0.39	0.27	0.29	0.39	0.15	0.37
Foursquare Venues	0.29	0.38	1.00	0.02	0.05	0.08	0.22	0.12	0.14	0.24	0.05	0.21
Twitter Tweets	0.11	0.05	0.02	1.00	0.02	0.01	0.08	0.19	0.18	0.07	0.03	0.02
OSM POI	0.33	0.13	0.05	0.02	1.00	0.03	0.32	0.54	0.52	0.20	0.63	0.04
OSM Roads	0.08	0.14	0.08	0.01	0.03	1.00	0.26	0.17	0.15	0.09	0.14	0.03
Schools	0.50	0.39	0.22	0.08	0.32	0.26	1.00	0.71	0.72	0.38	0.43	0.12
GRUMP	0.55	0.27	0.12	0.19	0.54	0.17	0.71	1.00	0.92	0.45	0.52	0.07
WorldPop	0.57	0.29	0.14	0.18	0.52	0.15	0.72	0.92	1.00	0.44	0.52	0.10
Landuse Urban	0.38	0.39	0.24	0.07	0.20	0.09	0.38	0.45	0.44	1.00	0.16	0.19
GeoNames Places	0.27	0.15	0.05	0.03	0.63	0.14	0.43	0.52	0.52	0.16	1.00	0.03
NE Pop. Places	0.29	0.37	0.21	0.02	0.04	0.03	0.12	0.07	0.10	0.19	0.03	1.00

We then calculate the *F*-score for each predictor against the FTP. *F*-score measures the relationship between the precision and recall of these datasets (Equation (1)). *Precision*, in this case, is the number of FTP locations correctly identified divided by the total number of locations identified whereas *recall* is the number of FTP locations correctly identified divided by the total number of actual FTP locations.

(1) $$F_{1}=2\cdot \frac{\text{precision}\cdot \text{recall}}{\text{precision}+\text{recall}}$$

**Fig. 3:**
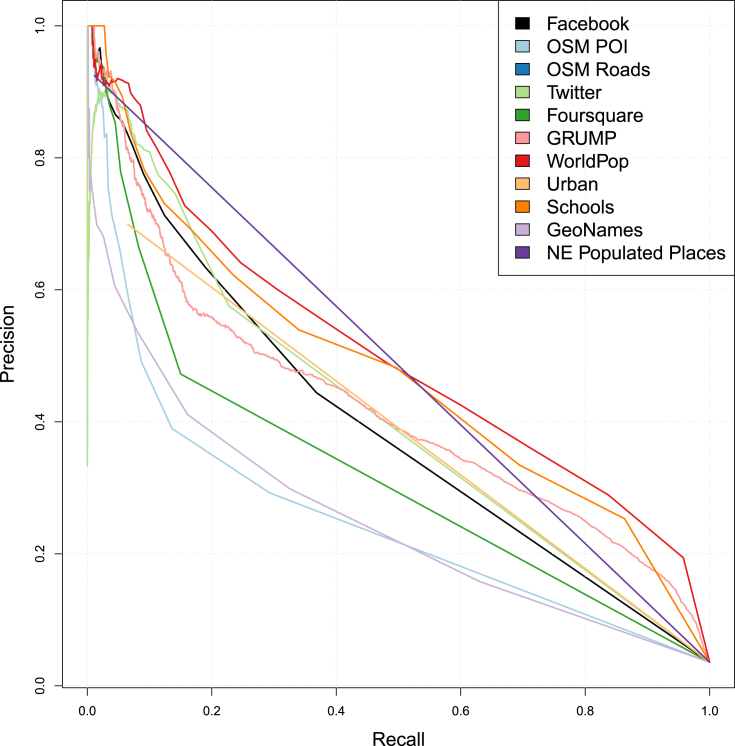
Precision vs. recall graphs for all independent variables.

Assessing the accuracy of a predictor via the *F*-score involves a trade-off. Fig. 3 shows precision versus recall for each of the predictor variables. Notably, the authoritative datasets show a steeper decrease in recall as precision drops below 0.4 whereas the user-contributed datasets tend to show fairly low trade-offs between the precision and recall. The highest *F*-score of *0.49* is found with the WorldPop data and a low of *0.07* with the Natural Earth Populated Places location data (Table 4). While these *F*-scores in combination with the correlation matrix show that the predictor datasets are of value in estimating FTP locations, on their own they only correctly identify a limited number of FTP in Kenya.

**Table 4:** Maximum F-score values for each of the predictor variables independently.

Dataset	Max F-score
Schools	0.38
GRUMP	0.44
WorldPop	0.49
Landuse Urban	0.12
GeoNames Places	0.31
NE Populated Places	0.07
Facebook Places	0.40
Foursquare Venues	0.23
Twitter Tweets	0.33
OSM POI	0.29
OSM Roads	0.21

### 4.2 Weighted combination of variables

Provided the accuracy of the predictors independently, we next explore a number of methods for combining the predictors in order to better identify the location of financial touch points in Kenya. Specifically, to address RQ2 we test four approaches to FTP identification, namely ordinary least squares regression, spatial lag regression, support vector regression, and random decision forest. The purpose of examining all of these methods is to determine which approach most accurately predicts the location of known FTP and produces a model on which to base further investigation into unknown FTP locations. To be clear, the regression approaches produces probability values that are used to in a prediction task of FTP in a grid cell or not. These probability values are later used in the generation of a *prediction layer* for inclusion in a mobile data collection application.

#### 4.2.1 Ordinary least squares model

A standard linear regression model was executed as a first step to determine the impact of each independent variable (predictor dataset) on identifying FTP. The data were separated by category as shown in Table 1, namely VGI, SM, or AUTH. Linear regression models were constructed for each category independently as well as combined. The independent variables, coefficients, R^2^, and residual standard error (RSE) for each model are shown in Table 5. Regarding multicollinearity between the independent variables, we note some small changes in the regression coefficients as predictors are added to the model. The most notable change here is in the OpenStreetMap POI dataset changing to having a negative influence on FTP identification when combined with all other datasets. Similarly, we see the *tweets* dataset change from having a significant impact on the model to not longer being significant. We calculated the condition indices (condition number test), measures of ill-conditioning in the predictor matrices and found that the regression models did not have significant multicollinearity. The conditional index values for the respective regression models are 5.87 (AUTH), 1.53 (SM), 1.77 (VGI), 7.43 (Combined).

**Table 5:** Results of the OLS and Spatial Lag regression models with four combinations of predictor variables. All coefficients are significant (p<0.001) except for Twitter OLS* which is not significant and Twitter SLM* with p<0.05.

Dataset	OLS Model Coefficient	Spatial Lag Model Coefficients
**Authoritative Datasets (AUTH) Model**		
Schools	3.80E-02	6.09E-02
GRUMP	9.56E-02	4.76E-02
WorldPop	2.23E-01	1.77E-01
Landuse Urban	1.06E-02	7.59E-03
GeoNames Places	−4.58E-02	−3.45E-02
NE Populated Places	5.54E-02	5.70E-02
Spatial Lag (Rho)	NA	2.33E-01
	$R^{2}$0.412, RSE 4.32E-03	$R^{2}$0.425, RSE 4.265E-03
**Social Media Datasets (SM) Model**		
Facebook Places	1.58E-01	1.33E-01
Foursquare Venues	5.55E-02	5.24E-02
Twitter Tweets	1.41E-01	3.39E-02
Spatial Lag (Rho)	NA	5.48E-01
	$R^{2}$0.267, RSE 4.82E-03	$R^{2}$0.423, RSE 4.27E-03
**Volunteered Geographic Information Datasets (VGI) Model**		
OSM POI	3.54E-01	2.34E-01
OSM Roads	8.57E-04	4.28E-04
Spatial Lag (Rho)	NA	5.52E-01
	$R^{2}$0.116, RSE 5.29E-03	$R^{2}$0.285, RSE 4.76E-03
**Combined (All data) Model**		
Schools	2.78E-02	3.05E-02
GRUMP	9.25E-02	5.22E-02
WorldPop	1.94E-01	1.60E-01
Landuse Urban	2.00E-03	−8.92E-04
GeoNames Places	−9.83E-02	−8.23E-02
NE Populated Places	3.04E-02	3.21E-02
Facebook Places	9.55E-02	9.59E-02
Foursquare Venues	4.30E-02	4.38E-02
Twitter Tweets	3.08E-02*	−8.72E-03*
OSM POI	−6.13E-04	1.02E-01
OSM Roads	1.05E-01	−6.23E-04
Spatial Lag (Rho)	NA	2.49E-01
	$R^{2}$0.489, RSE 4.02E-03	$R^{2}$0.502, RSE 3.97E-03

The AUTH-based regression produced an R^2^ value of 0.412 with all coefficients being significant (P < 0.001). Based on the coefficients, the WorldPop density values had the highest positive influence on the dependent FTP variable with GRUMP data also showing a high value of influence. The GeoNames places dataset had a small, but negative influence on the model. The SM-based regression model produced a lower R^2^ value meaning that less of the known FTP locations could be explained by our place-based and geotagged social media data. All coefficients were deemed significant with Facebook places and Tweets producing larger positive coefficients than Foursquare venues. The VGI-based linear regression models produced the lowest R^2^ value with OpenStreetMap POI having a much larger influence on the model than OpenStreetMap Roads. Combining all independent variables in one OLS linear regression model produced the highest R^2^ value with all coefficients having a significant impact with the exception of tweets and the lowest residual standard error of the OLS models. As a first, but important, step, these results are encouraging and indicate that a combination of social media, VGI and authoritative data produce better results for predicting financial touch points than each data type independently.

#### 4.2.2 Spatial lag model

Using the *Jarque-Bera* test (Jarque & Bera, 1980), the variables in the OLS models were assessed for normality of the distribution of errors. All probability values for the tests were very low indicating non-normal distribution of the error terms. Our next step was to geospatially map the residuals of our best-fit linear regression model in order to test for spatial autocorrelation in our predictors. Visually, the residuals appeared to show a clear spatial pattern with underestimation occurring near major cities such as Nairobi and overestimating in more rural regions to the North. *Moran's I* analysis of the residuals supported this assessment with significant global values of 0.305, 0.266, and 0.100 for SM, VGI, and AUTH models respectively, with a distance threshold of 0.02° (distance to the nearest grid cell). *Local Moran's I* analysis also found highly significant spatial clustering around the high density FTP regions, predominantly major cities. These results, combined with low probability values from Breusch-Pagan tests (Breusch & Pagan, 1979) for heteroskedasticity indicate a need to account for spatial autocorrelation in our regression analysis.

A spatial lag (Anselin, 2013) regression model (Equation (2)) was constructed relying on a Euclidean distance weighted matrix using Queen contiguity at a threshold of 0.02°. *Y* represents the vector of response variables, ρ the coefficients of spatial regression terms, making *WY* the spatial lag weighted response. *X* is the matrix of independent predictors, β the coefficient matrix of *X* and ε the random error vector. The results of the Spatial Lag regression models for the 3 groups of predictor variables and the combined model are shown in Table 5.

(2) Y=ρWY+βX+ε

In all cases, there was an increase in the amount of variance explained (R-squared) over the OLS regression models, and a relative decrease in the standard error of the residuals. The WorldPop population dataset still had a large influence in the combined dataset model (based on the coefficient value) while Tweets remained low in contribution and significance. The spatial lag (Rho) coefficients all had significant impacts on the respective models demonstrating that accounting for spatial dependency in such a model positively influenced the ability to predict FTP in Kenya. These results again indicate that combining datasets from various user-generated and authoritative sources positively influence the ability to predict FTP and that the inclusion of a spatial lag term positively contributes to an explanation of the variance in our model.

#### 4.2.3 Support vector regression

Support vector machine (SVM) analysis takes a different approach to prediction than the previous two analyses. SVM is nonparametric and approaches regression through a kernel function (Cortes & Vapnik, 1995; Drucker, Burges, Kaufman, Smola, & Vapnik, 1997). To start, we used an epsilon (ε = 0.1) type of regression with a linear kernel.^10^ This approach attempts to find a separating hyper-plane between the two classes, in our cases occurrence of FTP in a grid cell or not, with a maximum gap between. In general, SV regression perform better with a higher number of dimensions, or predictor variables in our case, and really only if the combination of these variables almost certainly leads to a known FTP. In our cases, neither of these conditions hold true as the number of datasets (dimensions) is relatively small and based on our previous OLS and spatial lag analysis, the variance explained is low. While this form of analysis was tested on our dataset, it primarily acts as a first *comparison* step in a machine learning approach to this problem.

#### 4.2.4 Random decision forest

Random decision forests (RDF) (Ho, 1995) are an ensemble learning method for regression, in our case, that construct a set of decision trees for the purpose of prediction. An optimal threshold value for identifying the occurrence of an FTP or not in a grid cell is calculated. A random forest aims to correct for overfitting, known to happen in a standard decision tree approach (Friedman, Hastie, & Tibshirani, 2001).

**Fig. 4:**
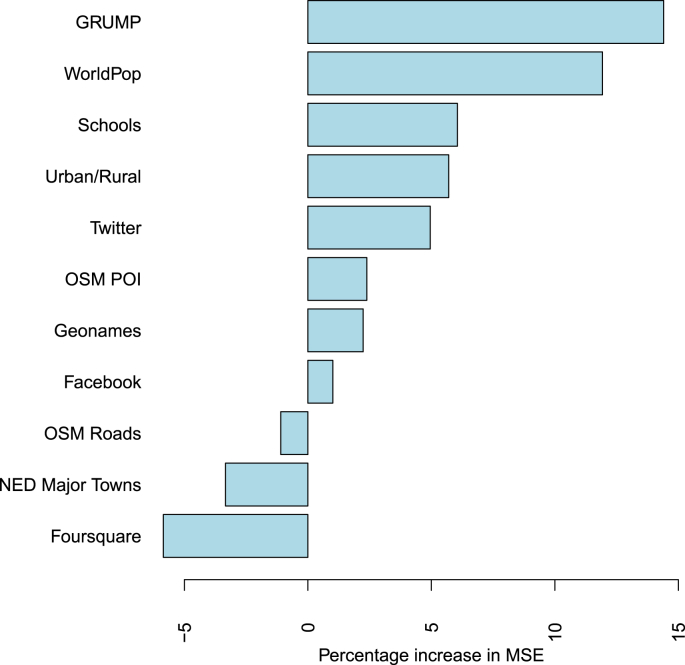
Percentage increase in mean square error of prediction as a result of variable shuffling. In essence, the higher the value, the more important that variable is to the RDF regression model.

The *R* RandomForest package^11^ was used to fit a random decision forest regression model to the FTP data based on each of the category predictor variables independently as well as all together. This resulted in a $1.39\times 10^{5}$ mean of squared residuals explaining 55% of the variance. This approach used 500 trees with 4 variables tried at each split. The incremental node purity for the model is shown in Table 6 and reports on the average change of impurities of a tree node (in which the variable was used) before and after a split. Plotting the percentage increase in mean square error (MSE) for the combined approach (Fig. 4) we find that many of the authoritative datasets are the most important to the regression fit. Tweets, OSM POI and Facebook places all positively contribute to the model, with OSM Roads, NE Populated Places and Foursquare venues having little impact on the RDF fit.

**Table 6:** Incremental Node Purity of the variables in the random decision forest model.

Dataset	IncNodePurity
GRUMP	2.32E-02
WorldPop	2.29E-02
Schools	2.22E-02
Landuse Urban	5.23E-03
Twitter	1.06E-02
OSM POI	9.62E-04
Geonames	4.27E-03
Facebook	1.69E-02
OSM Roads	5.98E-05
NE Major Towns	1.00E-03
Foursquare	4.75E-03

Given the known spatial dependency of the predictor variables (based on global and local Moran's I measures), we elected to construct a separate RDF model which included latitude and longitude coordinates as covariables. There is some evidence in the existing literature that the inclusion of geospatial variables in such a model can influence the accuracy of prediction (Cracknell & Reading, 2014). Given the non-parametric nature of RDF, these variables could be included in the model and used in the prediction assessment. This led to a slightly higher percentage explained variance (0.56 vs. 0.55) and latitude was found to be the second most important contributing variable as determined by the percentage increase in mean square error. Again, though the prediction method has changed substantially, the findings again support the fact that user-contributed data are important in location prediction.

## 5 Results

In this section we present the results of the analyses performed in the previous sections. Running each of the regression models (OLS, Spatial Lag, SVM, and RDF) with datasets from each of our categories (VGI, SM, AUTH) as well as a combination of all datasets (COMBO) produced a set of FTP prediction values for each cell in our Kenya grid, 16 different FTP prediction grids. These *regression-based prediction grids* were each then compared to our known FTP grid and three measures of accuracy were calculated for each prediction. Table 7 shows a comparison of the four regression techniques used in this work along with values for assessing accuracy of prediction including maximum F-score, Spearman's Correlation and root mean square error (RMSE). The SVM and RDF methods also show results for regression models that included all predictor variables as well as latitude and longitude centroids of the grid cells.

**Table 7:** Prediction results of the regression methods split by category of dataset. The maximum F-score, Spearman's Correlation and root mean square error are reported. Note that all Spearman correlation values are significant (p<0.01).

Method	Category	Max F-Score	Correlation	RMSE
OLS	VGI	0.31	0.340	5.29E-03
	SM	0.43	0.516	4.82E-03
	AUTH	0.49	0.678	4.32E-03
	COMBO	0.51	0.699	4.02E-03
Spatial Lag	VGI	0.36	0.429	5.13E-03
	SM	0.42	0.518	4.82E-03
	AUTH	0.49	0.637	4.34E-03
	COMBO	0.51	0.694	4.05E-03
SVM	VGI	0.28	0.301	5.35E-03
	SM	0.35	0.417	5.17E-03
	AUTH	0.47	0.582	5.21E-03
	COMBO	0.55	0.590	5.17E-03
	COMBO & LL	0.56	0.587	5.17E-03
RDF	VGI	0.31	0.606	4.69E-03
	SM	0.43	0.849	3.20E-03
	UGC	0.46	0.855	3.32E-03
	AUTH	0.57	0.930	2.25E-03
	COMBO	0.62	0.955	1.85E-03
	COMBO & LL	0.74	0.960	1.79E-03

In general, the random decision forest regression model approach produced the best results across most categories. The RDF model that included variables of all data categories, including latitude and longitude (LL) coordinates, produced the most accurate predictions as reported across all three measures. A maximum F-score of 0.74 is quite high considering the multitude of factors that may contribute to establishing an FTP. Similarly, a Spearman's correlation of 0.96 is extremely high but should by understood in the context of the sparsity of the FTP locations and predictions (most grid cells are 0). Lastly, the reported RMSE is low relative to the comparable RMSE values from all other methods and data categories.

**Fig. 5:**
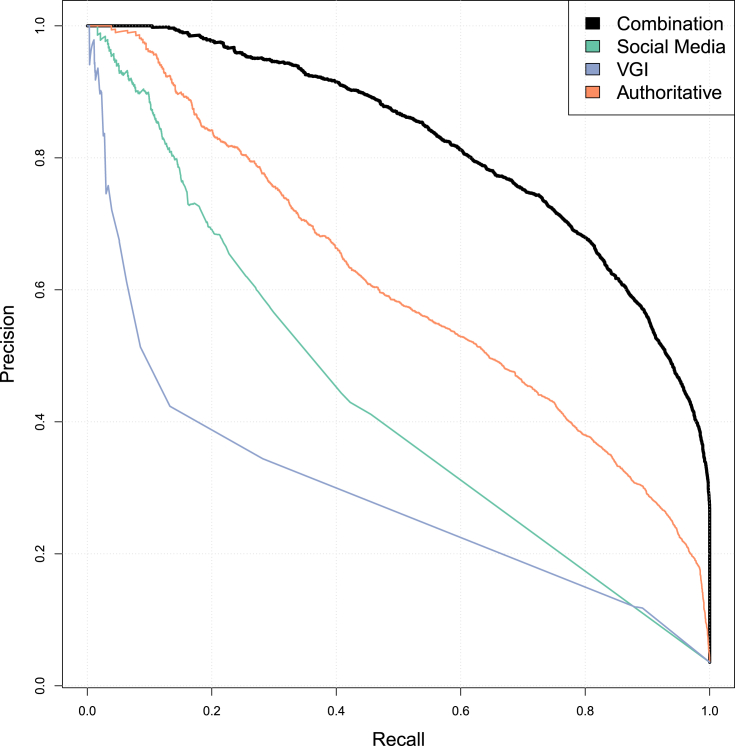
Precision vs. Recall for Kenya RDF predictions.

Fig. 5 further explains the F-scores for highest performing RDF model by plotting precision versus recall for the random decision forest models split by data category. In comparison to Fig. 3, the combined approach of all datasets produces a much better trade-off between precision and recall, specifically addressing RQ2 as stated in the introduction.

Next, the residuals of the best-fit RDF regression model are mapped back to the location data. Visual inspection identifies very little clustering within the residuals and a Moran's I analysis confirms this with a bootstrapped observed value of less than 0.001 implying a high degree of spatial randomness in these RDF-based residuals.

## 6 Validation

### 6.1 Ground-truthing in Kenya

One primary goal of this work was to build a prediction model that would allow researchers in the field to identify previously unidentified FTP in Kenya. With this goal in mind we used the best fit random decision forest model (reported in the previous section) to predict FTP locations across Kenya. The predicted number of FTP locations was subtracted from the previously known number of FTP per cell to produce a residuals map showing the difference between known and predicted FTP. Of these residual cells, we further investigated 47 that contained no known FTP and showed large negative values (indicating high probability of finding FTP). Identifying these locations with high potential is important as a single, previously unknown, FTP could potentially be servicing a number of inhabitants; Inhabitants that were thought to be without access to financial services.

**Fig. 6:**
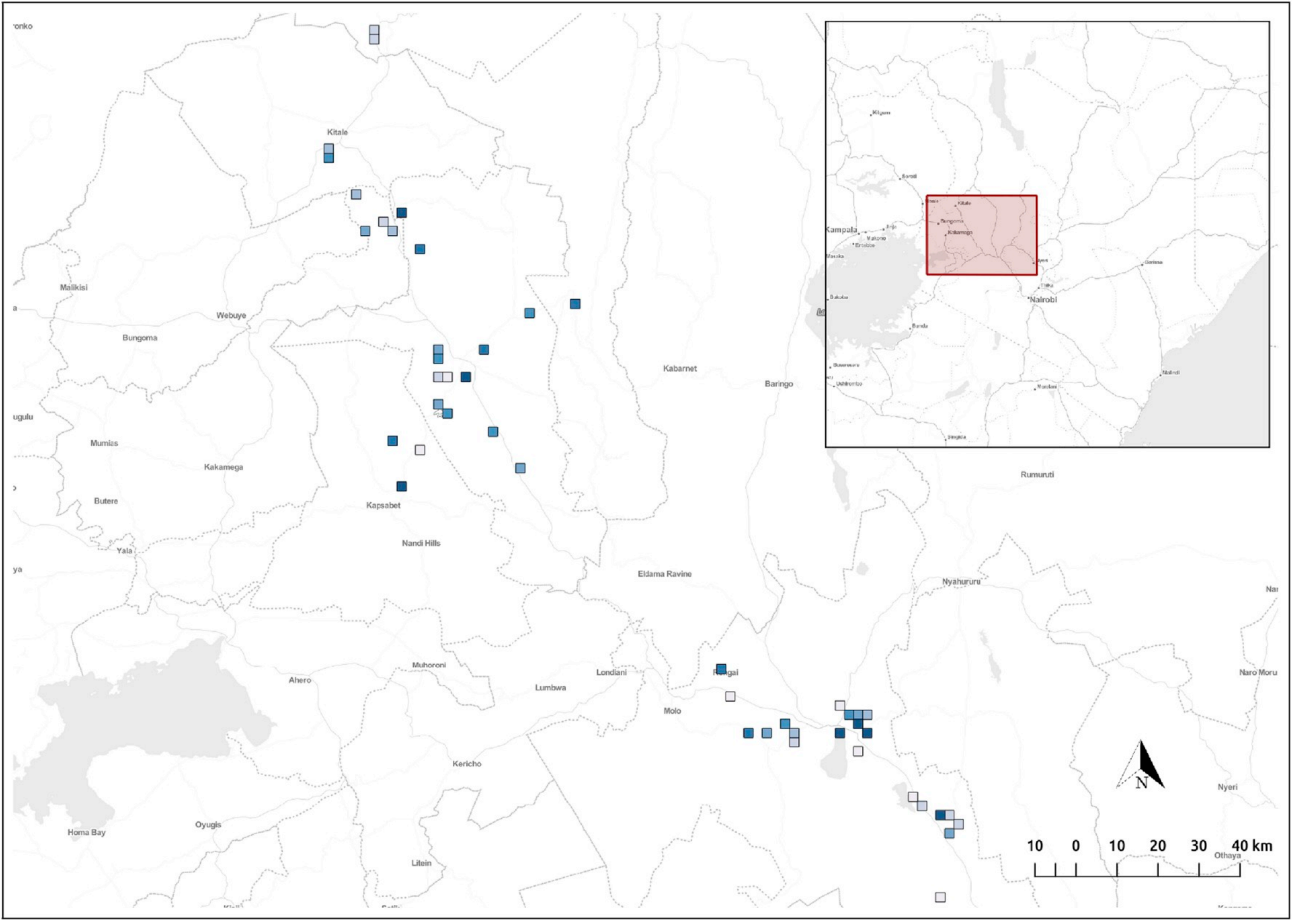
Previously unknown FTP location (47) identified by the prediction model. Blue color density indicates rank based on probability of finding at least one FTP within 1.1 km of the marked location. (For interpretation of the references to color in this figure legend, the reader is referred to the Web version of this article.)

These 47 potential FTP cells were ranked based on the size of the residual and the latitude and longitude coordinates of the centroids were shared with researchers on the ground in Kenya (see Fig. 6). The selection of these specific locations was also based on availability of data collection personnel in the region around Eldoret city in eastern Kenya. Data collectors traveled to these *high-FTP-potential* locations and recorded the presence and location of any FTP they found within 1 km radius of the cell centroid (represented as square markers in Fig. 6). In essence, the data collectors used the ranking of residuals for binary classification (decision to travel to location or not) and then counted the total number of FTP found within the vicinity of the marked location. In total, 203 previously unidentified FTP were recorded within the vicinity of these locations. In total, 41 of the 47 locations reported at least one previously unknown FTP location within a 1.1 km radius. Assigning the count of identified FTP to their nearest marked location (again, see Fig. 6) allowed us to compute the correlation between estimated FTP potential and count of actual FTP identified. The resulting Spearman's correlation was 0.233 (p<0.01), a small but positive correlation indicating that the magnitude of the residuals, not just the binary threshold, have a role to play in FTP identification. It should be noted that a 1.1 km cell radius is quite a large distance to explore and while quite a few new FTP were identified, it is likely that other FTP may existed in the area but were not identified.

The identification of these previously unidentified FTP offers validation to the RDF machine learning approach suggested in this research, and addresses RQ3. This approach presents a data-driven based method for uncovering previously unidentified FTP locations and has the potential to significantly reduces the on-the-ground efforts of individuals that previously relied on qualitative assessment and brute force search methods.

### 6.2 Applicability to neighboring countries

In order to test the limits of our RDF prediction approach, the best-fit regression model constructed from numerous datasets in Kenya was applied to datasets collected in the neighboring country of Uganda. The countries of Kenya and Uganda, while similar in many ways, also differ substantially. We are currently in the process of collecting further on-the-ground data to test the transferability of this model to the neighboring country of Uganda.

**Fig. 7:**
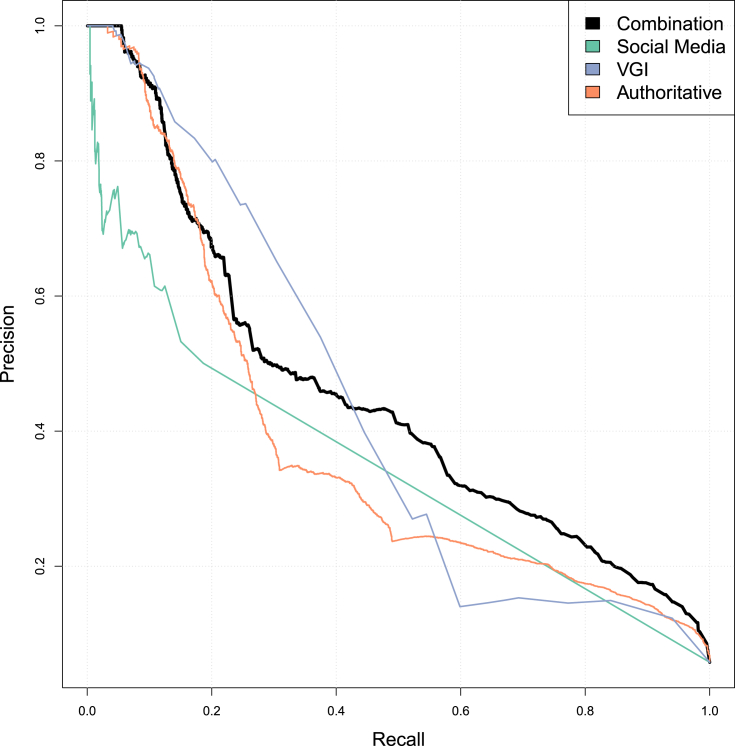
Precision vs. recall for Uganda RDF Predictions.

In the mean time, our naive approach was again to rely on the same publicly available datasets and use the best-fit model from the Kenya data to predict locations and number of FTP in Uganda. Fig. 7 graphs the precision versus recall for three data categories independently as well as the combined RDF regression model. Not surprisingly, the RDF model trained on Kenya data produces poorer results in Uganda than Kenya. The F-scores for the three data categories of SM, VGI and AUTH are 0.43, 0.44 and 0.36 respectively with a combined F-score of 0.44. The best Spearman's correlation value was 0.69 for the combined model with a RMSE of *6.08E-03*. In fact, just using OpenStreetMap POI data produced accuracy values (F-score, Correlation and RMSE) similar to the combined RDF model built from Kenya data.

There are numerous reasons for the drop in accuracy scores compared to Kenya. The most obvious answer is that these are different countries with unique economic, information & communications technologies (ICT), and socio-demographic properties. It is naive to assume that a model built on data from one country could be applied to a completely different country without a loss of accuracy. Second, the FTP location data were collected and reported by a different provider in Uganda than in Kenya (Humanitarian OpenStreetMap vs. Brand Fusion). There are likely differences in the data collection techniques, number of people involved and technology employed. Future work will explore these differences with the purpose of identifying key ways in which a model can be altered to account for regional differences.

## 7 Mobile application

One of the outcomes of this research, and the focus of RQ4, is an Android-based mobile application for identifying, creating, editing and deleting financial touch points within sub-Saharan Africa. The current prototype application functions both with and without a stable Internet connection and currently focuses on Kenya.

### 7.1 Prediction overlay

**Fig. 8:**
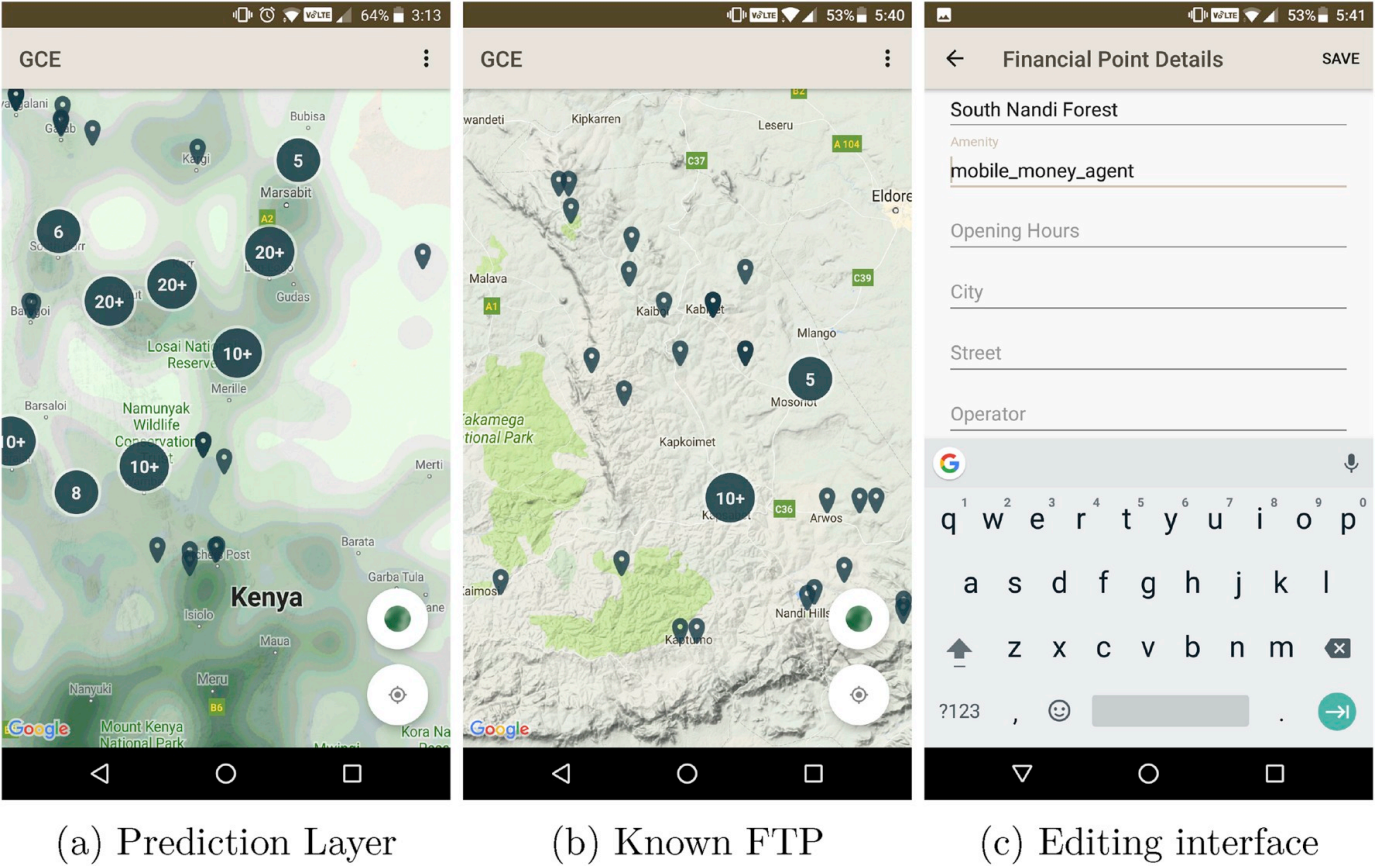
The FTP mobile prediction and capture application.

Based on the best-fit RDF prediction model developed in Section 4.2.4, a raster layer containing FTP location predictions was constructed at a resolution of 0.02°. This raster layer was styled on a white to green color ramp using natural break classification and tiled to allow efficient data transfer and visualization on the mobile mapping application (Fig. 8a).

### 7.2 Financial touch point locations

Upon loading, the mobile application prompts the user to download known FTP locations for one or more of Kenya's 70 districts. The purpose of this is to allow a user to download only the data required, thus reducing data usage and device storage. Before leaving an area of stable connectivity, the user will download the known FTP locations for the district(s) in which they will be traveling.

Users are invited to zoom and pan the map as they would on any standard mobile mapping application (Fig. 8b). The FTP locations are shown as point markers on the map and clustered depending on zoom scale. When the user selects a marker on the map, they are presented with the *Details* interface. This interface shows information collected about the FTP by the original party. The user can choose to edit this information (Fig. 8c) or delete the FTP entirely. Finally, the user has the option of zooming into their current location on the map, either through panning/zooming or selecting the *locate me* button. Once the map is at a reasonable scale, the user can tap the map to add a new FTP. In this case, the unpopulated *Edit* interface is presented to the user. Once the user is finished editing, adding and deleting FTP, they have the option (selection from the context menu) to upload the changes to the database. Again, this allows for offline editing and reduces overhead of constant communication with the server whenever a FTP is edited. The application is currently in use by data collection teams in Kenya.

## 8 Conclusions & future work

In this work we present a novel approach to identifying financial touch points in Kenya through combined use of geosocial media data, volunteered geographic information, and authoritative geospatial datasets (RQ1 and RQ2). We showed that we can significantly increase the ability to identify FTP locations by including both spatial and platially tagged social media posts in our analysis. Current state-of-the-art machine learning techniques were compared to existing ordinary least squares and spatial regression models and it was shown that a random decision forest model using combined data from all three sources best identified existing financial touch points and can be used to identify the location of previously unknown FTP (RQ3). With this goal in mind, we developed a mobile application for on-the-ground data collection that uses the results of the RDF model as a geospatial estimation layer through which users are be better informed on where to locate FTP (RQ4).

The application is currently in use in Kenya and has aided in the identification of previously unknown financial touch points. Data collection done using this application (with the inclusion of the prediction layer) has the potential to substantially impact financial services in countries such as Kenya and Uganda. Provided detailed maps of access to financial services in sub-Saharan Africa, local government and international agencies are better informed when formulating policies and regulating financial services. The goal of this work is to facilitate this discussion by providing access to the most up-to-date geospatial data.

This analysis does come with some limitations. Given the country-level analysis that was executed, a trade off was made when determining the cell size for analysis. Increasing or decreasing this cell size would understandably impact the accuracy of the identification model. Access to known FTP locations is another limiting aspect of this type of analysis. Two different data sets were collected from two different organizations in two different countries. The methods of data collection varied and there is likely bias in how the data was collected (e.g, accessibility of roads, daylight restrictions,etc.). While these biases potentially impacted the final results of the analysis, they had little influence on the methods of analysis that were employed. A limitation of the validation approach lies in the lack of collected information related to true and false FTP negatives. Data collection teams in Kenya did not report on the lack of FTP in regions that were identified as not having FTP as it was not their primary mandate. Future data collection campaigns will aim to collect these data.

Future work in this area will continue to focus on refining the identification model through inclusion of additional datasets, updating known FTP locations, and feedback from on-the-ground data collection efforts. Though this work is primarily focused on leveraging the relationship between external datasets and FTP, the role of *nearby* FTP within a known touch point dataset could potentially have an impact on the identification of new FTP as well. Additionally, we are in the midst of assessing the accuracy of our existing model and refining new models based on data from neighboring countries in the region. Further examination of neighboring country-specific datasets will lead to a better understanding of the impact that socio-economics, demographics, ICT adoption, etc. have on the ability to successfully identify FTP locations at a broader scale.

## Footnotes

1 These include mobile money providers, brick and mortar banks, etc.

2 http://openstreetmap.org.

3 https://hotosm.org/projects.

4 http://www.brandfusion-africa.com/services/mobile-money.

5 Note that there is a community-based validation process in OpenStreetMap.

6 https://github.com/MorbZ/OsmPoisPbf.

7 https://www.instagram.com/developer.

8 https://dev.twitter.com/streaming.

9 https://developer.foursquare.com.

10 R package: https://cran.r-project.org/web/packages/e1071.

11 https://cran.r-project.org/web/packages/randomForest/.

## References

[bib1] Adams B., McKenzie G., Gahegan M. (2015). Frankenplace: Interactive thematic mapping for ad hoc exploratory search. Proceedings of the 24th international conference on world wide web.

[bib2] Anselin L. (2001). Spatial econometrics, A companion to theoretical econometrics 310330.

[bib3] Anselin L. (2013).

[bib4] Armstrong P., Wang Y. (2018). Is alibaba losing to tencent in China's trillion-dollar payment war.

[bib5] Asongu S.A., Nwachukwu J.C. (2016). The role of governance in mobile phones for inclusive human development in sub-saharan africa. Technovation.

[bib6] Balk D., Deichmann U., Yetman G., Pozzi F., Hay S., Nelson A. (2006). Determining global population distribution: Methods, applications and data. Advances in Parasitology.

[bib7] B. A. of Kenya (2016). State of Internet in Kenya 2016.

[bib8] Brand Fusion (2015). Financial inclusion research project - handbook.

[bib9] Brand Fusion (2015). Kenya multi sector GIS mapping project final report.

[bib10] Breusch T.S., Pagan A.R. (1979). A simple test for heteroscedasticity and random coefficient variation. Econometrica: Journal of the Econometric Society.

[bib11] Broxton P.D., Zeng X., Sulla-Menashe D., Troch P.A. (2014). A global land cover climatology using modis data. Journal of Applied Meteorology and Climatology.

[bib12] C. A. of Kenya (2016). Quarterly sector statistics report.

[bib13] Chesnokova O., Nowak M., Purves R.S. (2017). A crowdsourced model of landscape preference.

[bib14] Cortes C., Vapnik V. (1995). Support-vector networks. Machine Learning.

[bib15] Cracknell M.J., Reading A.M. (2014). Geological mapping using remote sensing data: A comparison of five machine learning algorithms, their response to variations in the spatial distribution of training data and the use of explicit spatial information. Computers & Geosciences.

[bib16] Deville P., Linard C., Martin S., Gilbert M., Stevens F.R., Gaughan A.E. (2014). Dynamic population mapping using mobile phone data. Proceedings of the National Academy of Sciences.

[bib17] Dillon B. (2012). Using mobile phones to collect panel data in developing countries. Journal of International Development.

[bib18] Dobson J.E., Bright E.A., Coleman P.R., Durfee R.C., Worley B.A. (2000). Landscan: A global population database for estimating populations at risk. Photogrammetric Engineering and Remote Sensing.

[bib19] Drucker H., Burges C.J., Kaufman L., Smola A.J., Vapnik V. (1997). Support vector regression machines. Advances in neural information processing systems.

[bib20] Economist Intelligence Unit (2014). M-pesa: Out of africa, into Romania.

[bib21] European Investment Bank (2016). Banking in sub-saharan Africa:recent trends and digital financial inclusion.

[bib22] Facebook People Insights (2017). Journeys of connectivity: How people in sub-saharan africa come online.

[bib23] Freire S., Kemper T., Pesaresi M., Florczyk A., Syrris V. (2015). Combining GHSL and GPW to improve global population mapping. Geoscience and remote sensing symposium (IGARSS).

[bib24] Friedl M.A., McIver D.K., Hodges J.C., Zhang X., Muchoney D., Strahler A.H. (2002). Global land cover mapping from modis: Algorithms and early results. Remote Sensing of Environment.

[bib25] Friedman J., Hastie T., Tibshirani R. (2001).

[bib26] FSD Kenya (2015). FinAccess geospatial mapping 2013.

[bib27] Gislason P.O., Benediktsson J.A., Sveinsson J.R. (2006). Random forests for land cover classification. Pattern Recognition Letters.

[bib28] Goodchild M.F. (2007). Citizens as sensors: The world of volunteered geography. Geojournal.

[bib29] Guo J., Bouwman H. (2016). An ecosystem view on third party mobile payment providers: A case study of alipay wallet. Info.

[bib30] Ho T.K. (1995). Random decision forests. Document analysis and recognition.

[bib31] Howard P.N., Parks M.R. (2012). Social media and political change: Capacity, constraint, and consequence. Journal of Communication.

[bib32] Hughes N., Lonie S. (2007). M-pesa: Mobile money for the “unbanked” turning cellphones into 24-hour tellers in Kenya. Innovations.

[bib33] Hu Y., Janowicz K., Couclelis H. (2017). Prioritizing disaster mapping tasks for online volunteers based on information value theory. Geographical Analysis.

[bib34] Jarque C.M., Bera A.K. (1980). Efficient tests for normality, homoscedasticity and serial independence of regression residuals. Economics Letters.

[bib35] Kaigwa M., Madung O., Costello S. (2015). Nendo 2014/15 social media trend report.

[bib36] Kim J. (2016). Reaching the rural regions in Kenya through mobile money. http://finclusionlab.org/es/node/519/.

[bib37] Kirui O.K., Okello J.J., Nyikal R.A., Njiraini G.W. (2013). Impact of mobile phone-based money transfer services in agriculture: Evidence from Kenya. Quarterly Journal of International Agriculture.

[bib38] Linard C., Gilbert M., Snow R.W., Noor A.M., Tatem A.J. (2012). Population distribution, settlement patterns and accessibility across Africa in 2010. PLoS One.

[bib39] Linard C., Tatem A., Stevens F.R., Gaughan A., Patel N.N., Huang Z. (2014). Use of active and passive vgi data for population distribution modelling: Experience from the worldpop project.

[bib40] McKenzie G., Janowicz K. (2014). Coerced geographic information: The not-so-voluntary side of user-generated geo-content. Eighth international conference on geographic information science.

[bib41] McKenzie G., Janowicz K. (2015). Where is also about time: A location-distortion model to improve reverse geocoding using behavior-driven temporal semantic signatures. Computers, Environment and Urban Systems.

[bib42] Moran P.A. (1950). Notes on continuous stochastic phenomena. Biometrika.

[bib43] Natural Earth (2014). Populated places. http://www.naturalearthdata.com/downloads/10m-cultural-vectors/10m-populated-places/Ω.

[bib44] Ndung'u N. (2014). Understanding and expanding financial inclusion in Kenya.

[bib45] Ochieng M. (2016). The new money lenders of nairobi. https://www.fsdafrica.org/knowledge-hub/blog/the-new-money-lenders-of-nairobi/.

[bib46] Okolloh O. (2009). Ushahidi, or testimony: Web 2.0 tools for crowdsourcing crisis information. Participatory Learning and Action.

[bib47] Olingo A. (2016). Kenya, Uganda in plans to pull informal sector into tax bracket.

[bib48] Palen L., Soden R., Anderson T.J., Barrenechea M. (2015). Success & scale in a data-producing organization: The socio-technical evolution of openstreetmap in response to humanitarian events. Proceedings of the 33rd annual ACM conference on human factors in computing systems.

[bib49] Perez S. (2013). Foursquare begins crowdsourcing local business data collection with questions that appear after check-ins.

[bib50] Porter G. (2012). Mobile phones, livelihoods and the poor in sub-saharan africa: Review and prospect. Geography Compass.

[bib51] Rahemtulla H., Kaplan J., Gigler B.-S., Cluster S., Kiess J., Brigham C. (2012). Open data Kenya: Case study of the underlying drivers, principal objectives and evolution of one of the first open data initiatives in Africa.

[bib52] Ripley B.D. (1976). The second-order analysis of stationary point processes. Journal of Applied Probability.

[bib53] Ruktanonchai N.W., DeLeenheer P., Tatem A.J., Alegana V.A., Caughlin T.T., zu Erbach-Schoenberg E. (2016). Identifying malaria transmission foci for elimination using human mobility data. PLoS Computational Biology.

[bib54] Sekabira H., Qaim M. (2017). Can mobile phones improve gender equality and nutrition? Panel data evidence from farm households in Uganda. Food Policy.

[bib55] Shapshak T. (2017). Facebook has 170 million african users, mostly on mobile.

[bib56] Song C., Kwan M.-P., Song W., Zhu J. (2017). A comparison between spatial econometric models and random forest for modeling fire occurrence. Sustainability.

[bib57] Stefanidis A., Crooks A., Radzikowski J. (2013). Harvesting ambient geospatial information from social media feeds. Geojournal.

[bib58] Stevens F.R., Gaughan A.E., Linard C., Tatem A.J. (2015). Disaggregating census data for population mapping using random forests with remotely-sensed and ancillary data. PLoS One.

[bib59] Suri T. (2017). Mobile money. Annual Review of Economics.

[bib60] Suri T., Jack W. (2016). The long-run poverty and gender impacts of mobile money. Science.

[bib61] Tanle A., Abane A.M. (2017). Mobile phone use and livelihoods: Qualitative evidence from some rural and urban areas in Ghana. Geojournal.

[bib62] Triki T., Faye I. (2013). Financial inclusion in Africa.

[bib63] Uithol P. (2015). Mapping financial inclusion in Uganda.

[bib64] Wesolowski A., Buckee C.O., Bengtsson L., Wetter E., Lu X., Tatem A.J. (2014). Commentary: Containing the ebola outbreak-the potential and challenge of mobile network data. PLoS Currents.

[bib65] World Bank (2015). The global findex database 2014: Measuring financial inclusion around the world. Policy research working paper 7255.

[bib66] Xylouris A. (2016). Connected consumer survey 2016: Mobile services and devices in sub-saharan Africa.

